# Progress in Laryngology

**Published:** 1895-10-19

**Authors:** 


### PROGRESS IN LARYNGOLOGY.

(*Continued from page* 32.)

**Thyroid Gland.**—Fibrosis of this gland is a rare disease. An interesting case in point was shown at the May meeting of the Laryngological Society of London by Mr. Walter Spencer.[8] Commencing some years previously as an ordinary bronchocele, the latter grew gradually smaller and harder, so that when advice was sought, the thyroid was normal in size but of stony hardness. The pulse was 130, but there was no exophthalmos. The isthmus and portion of the adjacent lobes were removed to relieve dyspnœa, but the cartilaginous rings of the trachea were found to be absorbed by the pressure, and tracheotomy had therefore to be performed to procure the necessary relief. On section after removal, there was seen every gradation from normal tissue externally, to complete fibrosis towards the centre. Sufficient active tissue was left at the operation to prevent myxœdema, and the patient soon discarded the tube.

**Incipient Basedows Disease.**—Harrison Allen[9] has been noting certain clinical facts in the early history of enlargement of the thyroid, associated with throbbing of the carotids, and with some form of neurosis of the upper respiratory tract, but all apparently originating in a sudden shock to the system. He designates the group of symptoms by the above title for convenience, and considers them common, though never yet properly defined. He illustrates his point by narrating three cases, all of whom were female patients of more or less neurotic habit. The subject forms part of an interesting paper on "Age and Sex in Diseases of the Upper Respiratory Tract."

**Larynx.—Pulsus Paradoxus in Acute Laryngitis.**—In a case at the Manchester Infirmary, described by Dr. Edward Brockbank,[10] this symptom was well marked. The smaller beats occurred synchronously with inspiration, and the larger with expiration. Breathing at this time was extremely laboured, and the little patient was cyanosed. After tracheotomy, by which the symptoms were relieved, the pulse tracing became normal. At the autopsy membranous inflammation was found to extend from the larynx to the bronchi.

**Abductor Paralysis of the Vocal Cords.**—This is usually spoken of in text-books as a rare affection, though, thanks to the laryngological societies, one can see and study them much oftener than formerly. Rangé of Challes[11] speaks of it, however, as a very common motor disturbance. It is generally considered due to central nerve degeneration, such as occurs in locomotor ataxia, but Rangé states that many authorities now regard it as a manifestation of complete contracture, and proposes to describe it as a permanent adduction of the glottis, a term which presents the advantage of merely expressing the clinical aspect of the mischief, while being non-committal as to its cause.

**Benign Growths in the Larynx.**—Several interesting points were touched upon by Dr. Semon in his able lecture under this title,[12] delivered at St. Thomas's Hospital, on June 15th, 1895. He does not think the prevalent belief that chronic laryngeal catarrh is a prolific source of innocent growths, is founded upon trustworthy evidence; the catarrh is probably rather the consequence than the cause. Over use and misuse of the voice predispose to "singers' nodes" (chorditis tuberosa), but not to the production of true neoplasms. Of the many forms of histological structure met with, in the latter, perhaps the most curious and most recently described is that consisting of normal thyroid tissue; "attributable doubtless to hyperplasia of aberrant particles of thyroid gland." Fully 39 per cent. of benign growths are papillomata; next in order of frequency come fibromata, and next, but much more rarely, cysts. The commonest seats of papillomata, solitary or multiple, are the anterior thirds and commissure of the vocal cords. Those occurring elsewhere, except on the ventricular bands and in the ventricle, are to be regarded with suspicion. The same remark applies to such papillomata as being pointed and very white, and resembling a frosting of the surface rather than a cauliflower excrescence in miniature, are really malignant, and grow from an epitheliomatous basis. As regards symptoms, taking, for instance, the principal one, vocal disturbance, the rule has to be borne in mind that the higher up in the commissure the growth is attached the greater this will be. Everybody knows Dr. Semon's views as to the question of malignant degeneration of benign laryngeal growths, particularly with reference to operative interference. Whilst fully alive to the theoretical possibility of such a change, he denies any special liability, as well as the frequency of such an occurrence.

Spontaneous expulsion of new growths has been reported, but is of extreme rarity. Removal by the forceps is the only treatment worth consideration, and that by the endo-laryngeal method; the external operation is limited practically to sessile growths in the subglottic space, or even lower down. In a few cases treatment is neither required nor advisable. Dr. Dundas Grant's safety laryngeal forceps are a valuable acquisition for the removal of many forms of growths situated on the cords.

In the multiple papillomata of children the intubation method is far inferior to Scanes Spicer's plan. This consists of operating under chloroform, and repeated mopping of the pharynx and larynx, until secretion being completely arrested, the growths are capable of being seen and removed in the usual way.

**Recurrent Papilloma.**—An interesting case in which portions of growth were repeatedly removed endolaryngeally, having the typical appearance of simple papilloma, but in which subglottic growth gradually manifested itself showing a transition to malignant disease, has been published by Dr. Wolfenden.[14] Laryngo-fissure was performed, and a quantity of epitheliomatous tissue removed, the thyroid cartilage itself being involved. The patient made a good recovery, but subsequently died from heart disease.

**Total Extirpation of the Larynx for Malignant Disease.**—Wolff[15] makes the following general statement of cases that had survived the operation some time. Four were living respectively thirty-two months, three and a half years, four and one-third years, and four and a half years after, without any signs of recurrence. Three had remained free from recurrence from four and a half to six years, and then died of intercurrent disease; three had died from intercurrent diseases between twenty and twenty-two months after operation, and without recurrence.

**Tubercular Tumours of the Larynx** deserve careful study on account of their similarity to benign neoplasms (papillomata, &c.), from which they cannot be positively diagnosed till microscopically examined after removal. Clarke[16] has collected the reports of forty-two cases. They are generally coexistent with distinct signs of pulmonary disease, and are in reality secondary to a primary focus situated in the lungs. Their favourite seats are the ventricular bands, vocal cords, and ventricles, but they may occur anywhere in the larynx. If it is decided to remove them, the cold snare should be employed, and lactic acid or the galvano-cautery applied to the site, as ulcers frequently form after their removal.

**Angio-keratoma of the Right Vocal Cord.**—An unique case has been described and reported upon (with microscopical analysis) by Dr. E. J. Moure, of Bordeaux.[17] The growth was sessile, the size of a millet seed, and occupied the central portion of the cord.

[1] Bull. de l'Acad. de Méd., April and May, 1895. [2] B. M. J., May 4, 1895. [3] Med. Week, Jan. 11, 1895. [4] Amer. Med. and Surg. Bull., Jan. 15, 1895. [5] Therap. Gaz., March 15, 1895. [6] B. M. J., June 1, 1895. [7] Amer. Med. and Surg. Bull., April 1, 1895. [8] Ann. of Surg., May, 1895. [9] Amer. Journ. Med. Sci., May, 1895. [10] B. M. J., March 11, 1895. [11] Med. Week, May 10, 1895. [12] Clin. Journ., Feb. 20, 1895. [13] Arch. für Laryngol. II, No. 1, p. 1. [14] Journal of Lar., Rhin., and Otol., March, 1895. [15] Amer. Journ. of Med. Sci., Feb., 1895. [16] Amer. Journ. of Med. Sci., May, 1895. [17] Ibid., Jan., 1895.

